# Horsetail (*Equisetum Arvense*) as a Functional Filler for Natural Rubber Biocomposites

**DOI:** 10.3390/ma13112526

**Published:** 2020-06-02

**Authors:** Marcin Masłowski, Justyna Miedzianowska, Agnieszka Czylkowska, Krzysztof Strzelec

**Affiliations:** 1 Institute of Polymer & Dye Technology, Lodz University of Technology, Stefanowskiego 12/16, 90-924 Lodz, Poland; justyna.miedzianowska@dokt.p.lodz.pl (J.M.); krzysztof.strzelec@p.lodz.pl (K.S.); 2 Institute of General and Ecological Chemistry, Lodz University of Technology, Żeromskiego 116, 90-924 Lodz, Poland; agnieszka.czylkowska@p.lodz.pl

**Keywords:** horsetail, *Equisteum arvense*, natural rubber, biocomposites

## Abstract

Over the past decades, increased scientific and research activity has been observed in the development of new, innovative materials for various end uses. This is mainly due to the growing ecological, environmental, and material awareness of many industries and societies. *Equisteum arvense*-horsetail is a plant that has demonstrated its properties in pharmacological and clinical aspects as well as in vitro and in vivo biological activity. This article presents a new method of using horsetail as a natural, lignocellulosic filler for a natural rubber matrix. In-depth characteristics of the applied bio-additive were prepared based on several research techniques and methods such as ultraviolet-visible spectroscopy, Fourier-transform infrared spectroscopy, scanning electron microscopy with energy dispersive X-RAY spectroscopy, thermogravimetric analysis, and flame atomic absorption spectroscopy. Elastomer composites were prepared as a function of horsetail content. Then, an analysis of their main functional properties was performed, including mechanical properties and susceptibility to accelerated aging processes such as thermo-oxidative, ultraviolet radiation, and weathering. The research emphasizes the significant value of horsetail in its new role—as an active filler of elastomer biocomposites. The obtained results confirmed that horsetail is lignocellulosic material thermally stable up to 180 °C. Horsetail is an active filler to natural rubber, positively affecting mechanical strength. Due to the presence of flavonoids and phenolic acids in horsetail, it can be used as a polymer anti-aging agent.

## 1. Introduction

*Equisetum arvense*, commonly referred to as horsetail, is a well-known and widespread pteridophyte growing in the northern hemisphere [1]. It occurs abundantly throughout Europe, Asia, and North America [2]. *E. arvense* is a species of perennial plant from the Equisetaceae family. It is the only contemporary genus belonging to the horsetail type (cluster) [2]. The history of Equisetum dates to the Cretaceous period, and it is estimated that perhaps even the Triassic. This suggests that it may be one of the oldest living types of vascular plants [3]. The Equisteum species has been the subject of increased research undertaken over the decades [4,5,6].

Horsetail has been used for centuries as a traditional, folklore medicinal plant for various types of aches and pains [2]. Current research has confirmed that Horsetail exhibits a lot of pharmacological applications as pain-relieving agent [7], hepatoprotective activity [8], treatment of anemia [9], antidiuretic activity [10], antimicrobial activity [1], herbal treatment for nail disorders, skin and hair remedy, relieving rheumatism pain, beneficial for cardiovascular problems, as well as its usefulness to promote the growth and stability of the skeletal structure [11].

The organic matrix of horsetail consists of cellulose that may exist in many polymorphs (cellulose I, II, III, and IV with additional subclassification); hemicellulose—heterogeneous polysaccharides found between lignin and cellulose fibers; lignin—phenolic polymeric material, pectin, waxes, essential oils, resins, and other water-soluble substances [12]. On the other hand, it contains more than 10% inorganic substances (two-thirds of which are silicic acid and potassium salts). In addition, horsetail is rich in sterols: β-sitosterol, campasterol, isofucosterol [13], ascorbic acid, phenolic acids (cinnamic acids, caffeic acid, di-E-caffeo-meso-tartaric acid and 5-O-caffeoylosimic acid), acids polyester, rare dicarboxylic acids (equizole acid), flavonoids [14], and styrylrons [15].

Horsetails are also known as biosilicates. Although the mechanism underlying the deposition of silica in these plants remains largely unknown, silica based on natural resources are of great interest in the field of material engineering and biomedicine due to the availability, low cost, and environmental friendliness of these materials [16,17]. Generally, biosilication can recently be defined as "movement of silicic acid from environments where its concentration does not exceed its solubility (<2 mM) to intracellular or systemic compartments in which it is collected for subsequent deposition as amorphous hydrated silica" [18]. Many living organisms, including higher plants, mollusks, sponges, and protozoa, have evolved to produce biogenic silica at an amazing rate of gigatons per year [19] and one of the best known of these is the horsetail.

Horsetail, due to many chemical components, among others, such as alkaloids, triterpenoids, flavonoids, phenols, and tannins [20], has been the subject of many scientific studies, mainly pharmaceutical. Its antioxidant, anti-inflammatory, anti-diabetic, anti-bacterial, anti-fungal, anticonvulsant, and anti-tumor activity has been confirmed [21,22,23].

The characteristic structure, chemical composition, and properties of horsetail make it easily classified as natural fiber fillers, as well as straw, jute, sisal, bamboo, coconut, sugar cane, etc. This material, combined with natural rubber (the first biopolymer with good elastic properties, resilience, and damping properties) can be a valuable additive to new composites with high application potential. Literature reports on the use of natural fiber reinforced rubber composites in the production of v-belts, hoses, tire treads, and complex-shaped mechanical goods are widely known [24]. Moreover, vulcanizates of the NR/horsetail type can show a significant advantage over currently produced composites due to the antioxidant potential of *Equisteum arvense*, which can extend the potential life of such products.

So far, however, there are no literature reports on the use of *Equisteum arvense* as an additive to polymer composites. Particularly interesting, from the point of view of its environmental aspect, is that it seems to be the addition of horsetail to the natural polymer, which is natural rubber. The purpose of the presented research is to accurately characterize the produced biocomposites, which can be a valuable contribution to the knowledge of the use of horsetail as a lignocellulosic material for elastomeric materials. Great hopes can be associated with the plant’s characteristics regarding active anti-oxidation components and the content of lignin and cellulose. Moreover, it is extremely important that the basic substrates are natural and renewable materials, which further emphasizes the ecological aspect and environmental friendliness of such composites. From the available literature there are no scientific articles devoted to natural rubber/horsetail composites. This fact is an additional advantage of highlighting the scientific novelty of the presented research.

## 2. Materials and Methods

### 2.1. Polymer

Natural rubber (NR) RSS I was obtained from Torimex-Chemicals Ltd. Sp. z o.o (Konstatntynów Łódzki, Poland). Rubber mixtures were cured using a conventional curing system containing sulfur (S) (Siarkopol, Tarnobrzeg, Poland) in the presence of zinc oxide (ZnO) (Sigma-Aldrich, St. Louis, MO, USA), stearic acid (SA) (Avantor Performance Materials, Gliwice, Poland), and 2-mercaptobenzothiazole (MBT) (Sigma-Aldrich, St. Louis, MO, USA).

### 2.2. Filler

Horsetail (HT) was provided by ManuTea (Chałupki, Poland). The herb was ground using a Pulverisette 5 Classic Line planetary ball mill (Fritsch, Idar-Oberstein, Germany) for 30 min, with a speed of 300 rpm. Next, the powder was dried at 70 °C to a constant weight.

Horsetail powder was tested in the application of UV–VIS diffuse reflectance spectroscopy using an Evolution 201/220 UV-Visible Spectrophotometer (Thermo Scientific, Waltham, MA, USA).

Fourier transform infrared (FTIR) spectrum of filler was recorded using a Nicolet 6700 FTIR spectrometer and OMNIC 3.2 software (Thermo Scientific, Waltham, MA, USA).

The thermal stability of filler was tested using Thermogravimetric Analyzer TGA (Mettler Toledo, Greifensee, Switzerland). The measurement was carried out in an argon atmosphere at temperatures from 25 to 700 °C with a heating rate of 10 °C/min.

The analysis of metal (II) concentration, was prepared for both pure horsetail and filled composites. Samples (each of about 20 mg) were digested in the mixture of concentrated acids (1 mL of 36% HCl and 6 mL of 65% HNO_3_) and decomposed using the Anton Paar Multiwave 3000 closed system instrument. Mineralization was carried out for 45 min at 240 °C under pressure 60 bar. The contents of Co(II), Ni(II), Cd(II), and Pb(II) in the samples were determined by the F-AAS spectrometer (Analytik Jena, Jena, Germany) with a continuum source of light and using air/acetylene flame. Absorbances were measured at analytical spectral lines: 240.7 nm for Co(II), 232.0 nm for Ni(II), 228.8 nm for Cd(II) and 217 nm for Pb(II). Standard solutions Merck (1000 mg/L) were used for the preparation of calibration curves.

The morphology of the horsetail surface and the tensile fracture surfaces of the natural rubber composites was analyzed by means of the SEM-EDS (Zeiss, Oberkochen, Germany), a LEO 1530 Gemini scanning electron microscope equipped with the energy dispersive spectrometer. The EDS method was used to obtain maps of elemental distribution on the micro area of filler. Before the SEM-EDS measurement, the samples were coated with a carbon target by using the Cressington 208 HR system. The particle size histogram was prepared using Image Processing and Analysis Java software—ImageJ 1.52p by Wayne Rasband (National Institute of Health, Washington, DC, USA) with MorphoLibJ plug-in by David Legland.

Rubber mixtures were prepared in a Brabender (Duisburg, Germany) measuring mixer N50 (temp., 50 °C; rotor speed, range 40 min^−1^; time of the process, 15 min). Then, two-roll mill was used to the addition of the vulcanization system and obtain blends sheets. The formulations of composites were shown in Table 1.

The rheometric properties and curing time of rubber mixtures were examined using a rotorless rheometer Model—MDR (Alpha Technologies, Bellingham, WA, USA) at 160 °C, according to standard ISO 6502.

The polymer mixtures were cured at 160 °C and 15 MPa pressure for a time corresponding with curing properties (t_90_). The specimens were formed using steel molds heated by an electrically hydraulic press.

The thermal stability of vulcanizates was tested with a TGA/DSC1 analyzer (Mettler Toledo, Greifensee, Switzerland). The weight loss of the samples at a heating rate of 10 °C/min was measured in argon (flow rate of 60 mL/min) in the temperature range of 25 °C–600 °C. Then, the measurement was continued in the air (60 mL/min) up to 900 °C.

The mechanical properties of natural rubber composites were conducted according to the standard procedures in ISO 37 on the universal testing machine Zwick (Roell Group, Ulm, Germany). The measurements were carried out at a crosshead speed of 500 mm/min. The test for each vulcanizate was repeated 5 times (before and after aging).

To determine the susceptibility of materials to accelerated aging processes, the composites were subjected to three different tests:

- the UV ageing process was simulated in a UV 2000 apparatus from Atlas (Mount Prospect, IL, USA). The conditions in the aging chamber included two repeating segments: daily segment (UV radiation intensity = 0.7 W/m^2^, temperature = 60 °C, duration = 8 h) and night segment (without UV radiation, temperature = 50 °C, duration = 4 h);
- the weather aging process was performed using a Weather-Ometer Ci 4000 (Atlas, Mount Prospect, IL, USA). Parameters of aging: radiation (340 nm) intensity = 0.4 W/m^2^, temperature = 38 °C, humidity = 55%;
- the thermo-oxidative degradation of the natural rubber biocomposites was carried out at a temperature of 70 °C for 14 days, according to the PN-82/C-04216 standard. Samples were exposed to air in a dryer (Binder, Tuttlingen, Germany) with thermo-circulation.

Based on changes in mechanical properties, the aging factor (K) was calculated according to Equation (1) [25]:

K = (TS × Eb)_after aging_/(TS × Eb)_before aging_ (1)

TS—tensile strength (MPa), Eb—the elongation at break (MPa).

Color stability during aging was tested using a CM-3600d spectrophotometer (Konica Minolta Sensing, Osaka, Japan). The measurements were carried out in the spectral range between 360 nm and 740 nm. The color test for each sample was repeated 5–7 times (before and after aging).

The total color change of the natural rubber vulcanizates was determined based on the following Equation (2) [26]:

(2) $$\Delta E=\sqrt{{\left(\Delta L\right)}^{2}+{\left(\Delta a\right)}^{2}+{\left(\Delta b\right)}^{2}}$$

where ΔL—the level of lightness or darkness, Δa—the relationship between redness and greenness and Δb—the relationship between blueness and yellowness.

The crosslinking density of the vulcanizates was determined by equilibrium swelling in toluene, based on the Flory-Rehner [27] Equation (3):

(3) $$\gamma_{e}=\frac{\ln\left(1-V_{r}\right)+V_{r}+\mu V_{r}^{2}}{V_{0}\left(V_{r}^{\frac{1}{3}}-\frac{V_{r}}{2}\right)}$$

$\gamma_{e}$—the crosslinking density (mol/cm^3^), V_0_—the molar volume of solvent (106.7 cm^3^/mol); µ—the Huggins parameter of the polymer-solvent interaction, was calculated from the Equation (4) [28]:

(4) $$\mu =\mu_{0}+\beta \cdot V_{r}$$

µ_0_—the parameter determine of non-crosslinked polymer/solvent relations, β—the parameter determine of crosslinked polymer/solvent relations (µ_0_ = 0.478, β = 0.228), Vr—the volume fraction of elastomer in the swollen gel (Equation (5)) [29]:

(5) $$V_{r}=\frac{1}{1+Q_{w}\frac{\rho_{r}}{\rho_{s}}}$$

$\rho_{r}$—density of rubber (0.99 g/cm^3^), $\rho_{s}$—density of solvent (0.86 g/cm^3^), Q_w_—the weight of equilibrium swelling:

(6) $$Q_{w}=\left(\frac{m_{\mathrm{sw}}-m_{s}}{m_{s}}\right)\cdot \left(\frac{100+x}{100}\right)$$

m_sw_—the weight of the swollen sample; m_s_—the weight of the dry sample; 100—the elastomer content in the sample; x—the filler content in the sample.

To determine the Q_w_ value, 4 samples were cut from each vulcanizate so that their mass was in the range of 20–50 mg. The samples soaked in toluene for 48 h (until equilibrium is reached). After this time, the samples were weighed (m_sw_—the weight of the swollen sample) and dried to constant weight at 50 °C for 48 h and then weighed (m_s_—the weight of the dry sample).

In the case of mechanical properties, crosslinking density and total color change of natural rubber vulcanizates a statistical analysis based on standard deviation was applied.

## 3. Results and Discussion

### 3.1. Ultraviolet–Visible Spectroscopy (UV-VIS)

**Figure 1:**
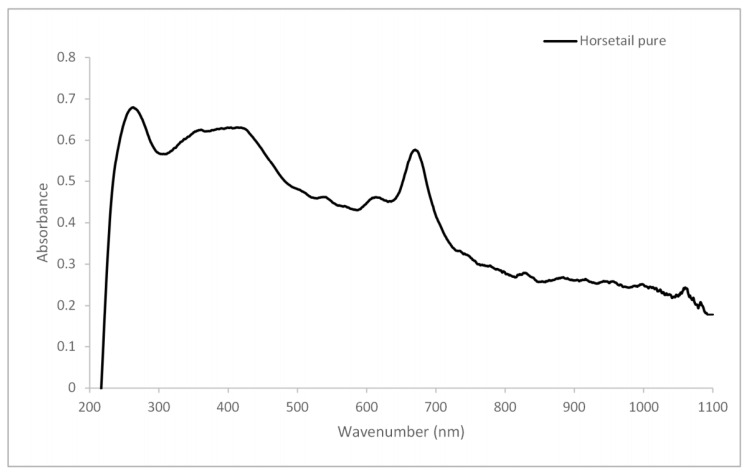
The UV-VIS spectrum of horsetail.

The UV-VIS spectrum of horsetail detected strong absorption peaks at 280–230 nm and 300–380 nm (Figure 1). This might indicate the presence of flavonoids and phenolic acids in the material. According to Masek [30], the maximum absorbance of flavonol occurs at 350 nm. Moreover, in the range of 280–230 nm, many phenolic acids contained in *Equisteum arvense* exhibit their absorbance maxima, e.g., ferulic acid, syringic acid, protocatechuic acid, vanillic acid, caffeic acid [31]. The absorption bands observed in the visible range were associated with the presence of chlorophyll in horsetail. Chlorophylls a and b exhibited maximum absorbance in the ranges 400–500 nm and 600–700 nm [32].

### 3.2. Thermogravimetric Analysis (TGA) of the Filler

**Figure 2:**
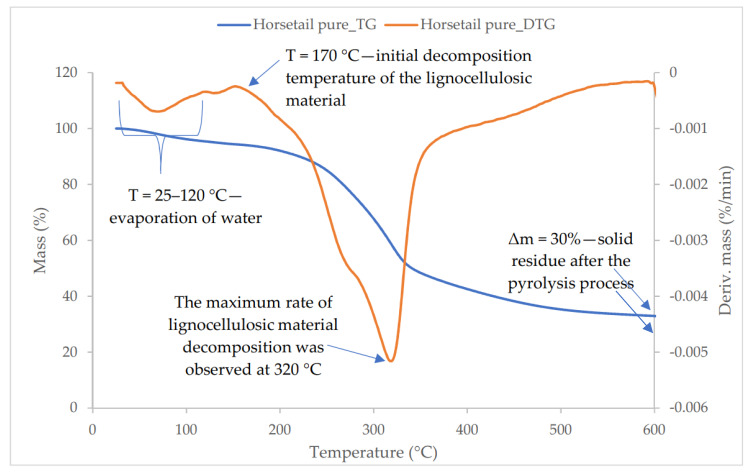
The thermogravimetry (TG) and derivative thermogravimetry (DTG) curves of the horsetail powder.

Figure 2 showed a thermogravimetric analysis of horsetail powder. The thermal decomposition of plant material can be divided into several weight loss stages, which were manifested by the presence of peaks on the DTG curve. In the first stage, the water contained in the material evaporated (T < 120 °C). The next stage could be associated with weight loss due to the decomposition of volatile oils (120–150 °C). Then, at a temperature of about 170 °C, the process of plant biomass degradation began, which resulted from the degradation of lignin, hemicellulose, and cellulose pyrolysis. According to Yang, in the wide temperature range between 170 °C and 500 °C occurs a long-lasted and slow degradation process of lignin. Other organic ingredients like hemicellulose and cellulose are thermally decomposed in at 220–315 °C and 315–400 °C, respectively [33]. Analysis of these three compounds (hemicellulose, cellulose, lignin) revealed lignin to be the most difficult one to decompose. Aromatic polyphenols found in the chemical structure of lignin affect its thermal stability, which results in a higher destruction temperature of lignin than cellulose [34]. The maximum rate of lignocellulosic material decomposition was observed at 320 °C (peak maximum on the DTG curve), at this temperature lignin and cellulose were most intensively decomposed. At higher temperatures (>400 °C) further slow decomposition of lignin could occur, which was inhibited at above 500 °C. The solid residue after the pyrolysis process was ~30%, with a high proportion of silica [35].

### 3.3. Fourier-Transform Infrared Spectroscopy (FTIR)

**Figure 3:**
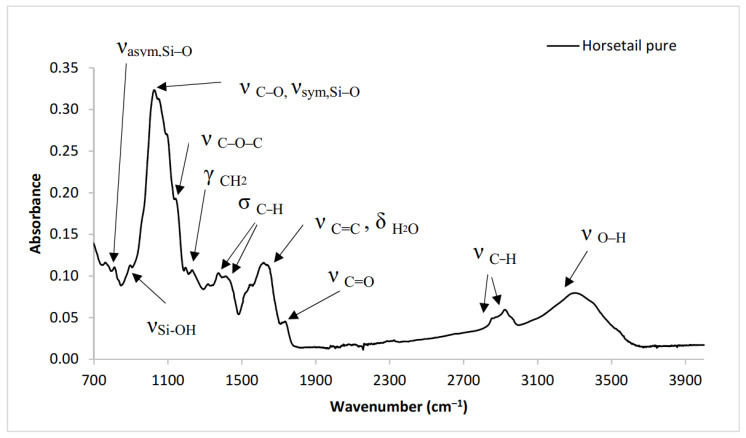
The FTIR spectrum of horsetail.

To determine the chemical structure of horsetail, ATR spectrum was performed (Figure 3). Analyzing the spectrum, a peak appeared in the 3600–3000 cm^−1^ absorption range, which was related to the stretching of OH bonds. Absorption bands with maxima at 2950 and 2850 cm^−1^ attributed to the CH aliphatic stretching of the methyl (CH_3_) and methylene (CH_2_) groups of lignocellulose material have also been observed [36]. A small peak with a maximum at ~1740 cm^−1^ was a confirmation of the presence in the chemical structure of carbonyl moieties (C=O) that occur in hemicellulose or waxes. A broad peak at ~1640 cm^−1^ might be associated with water absorbed by lignocellulosic material and/or the presence of aromatic and C=C functional groups. Such a structure occurs in high molecular mass aldehydes or ketones, waxes, fatty acids or esters [37]. The absorbance at 1410 and 1370 cm^−1^ are attributed to C–H stretching and C–H bending deformation, respectively [12]. 1010, 1030 and 1150 cm^−1^ peaks are characteristic of the C–O, C=C and C–O–C groups stretching of cellulose, hemicellulose and lignin [38]. The peaks at 1030 cm^−1^, 950 cm^−1^ and 800 cm^−1^ might correspond with the antisymmetric Si–O–Si, Si–OH and symmetric Si–O–Si stretching deformation, respectively [39]. These peaks could indicate the presence of silica in the material.

### 3.4. Analysis of Metals(II) Concentration

High concentrations of heavy metals have a negative effect on plant productivity, food quality, and environmental health. Even at low concentrations, they may be genotoxic, carcinogenic, mutagenic, and teratogenic in nature [40]. Plants accumulate both essential and toxic metals. Uptake of heavy metals and accumulation in plant tissues varies from environmental conditions, plant characteristics, and metal specificity [41]. The aim of this study was to assess the ecotoxicity effects of some heavy metals both in the used horsetail and natural rubber composites. In horsetail the concentrations of study metals(II) were low (Table 2). The nickel concentration was different significantly from the rest of the determined metals(II). This element is mineral nutrients for plants [42,43] and its concentration was within the generally accepted norm. In the case of natural rubber, the cadmium content was higher than for horsetail, while the cobalt and nickel contents were lower than for the plant. Relatively high lead concentration in natural rubber resulted from its processing. This concentration decreased with smaller amounts of added rubber in the mixture. In the case of cobalt and nickel concentrations for samples 10HT and 20HT a slight increase in concentrations was observed along with an increase in horsetail contents. For samples 30HT, 40HT, and 50HT, concentrations of cobalt and nickel were almost at constant levels. These metals belong to the elements necessary for the proper development of plants. They are microelements and their concentrations in various parts of plants could be different which slightly affected these data.

### 3.5. Morphology of the Filler and Biocomposites

**Figure 4:**
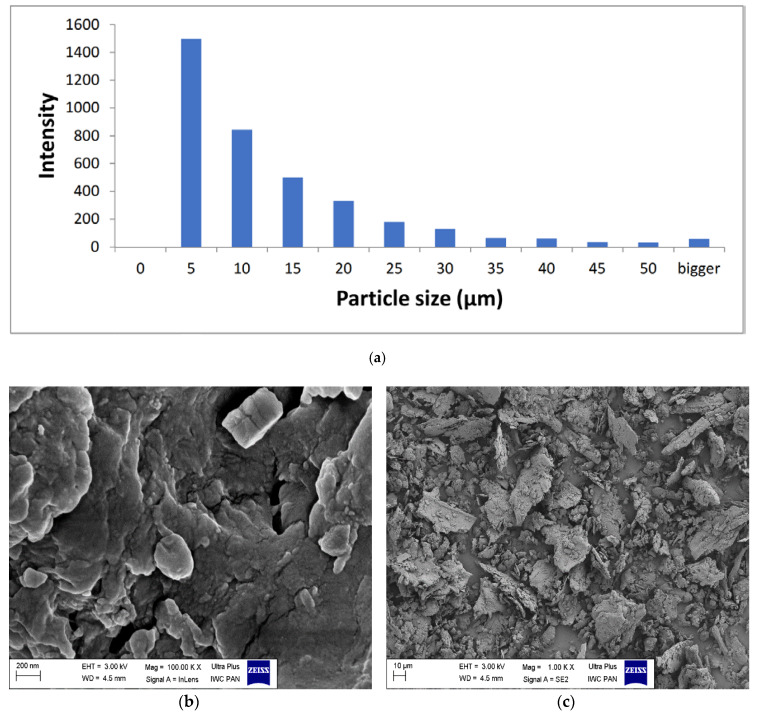
Histogram of the filler particle size (**a**) and SEM images of grounded horsetail under magnification: (**b**) 100.00× (**c**) 1.00×.

Figure 4 presents SEM images of horsetail powder at various magnifications (b) 100.00× and (c) 1.00×. It is noteworthy that the filler particles were different in size and shape. These particles were formed as a result of mechanical milling and were characterized by dimensions ranging from several hundred nanometers to several dozen microns (Figure 4a).

**Figure 5:**
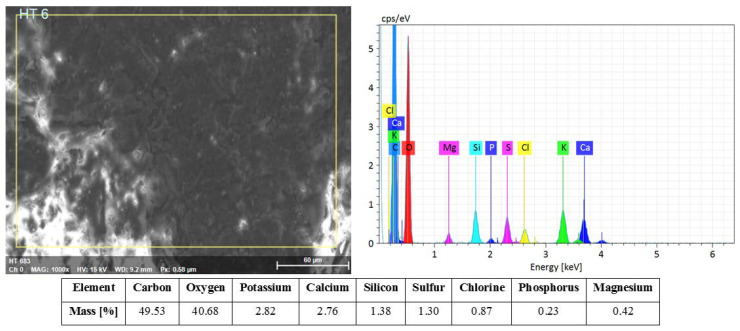
SEM-EDS analysis of horsetail.

The horsetail biomass consisted mainly of carbohydrates, which was confirmed by EDS analysis (Figure 5). However, the carbon content of the sample might be overstated and subject to error due to the pre-treatment of the material by carbon sputtering. It is estimated that carbohydrates account for over 70% of the mass of horsetail [44]. Among terrestrial plants, horsetail is one of the best-recognized one capable of accumulating silicon. EDS analysis of horsetail showed a high content of this element (Si) in the tested material (1.38%). Law et al. [45] proved that silicon is observed in all parts of *E. arvense*. Holzhuter et al. [46] investigated that Si forms thin layers on the inner surface of cell walls. Besides, it deposited as discrete knobs and rosettes. [47]. Other alkaline and alkaline earth elements such as magnesium (Mg), potassium (K), and calcium (Ca) were also found in the mineral deposits of horsetail. Moreover, it also contained elements such as aluminum, sulfur, chlorine, and phosphorus.

**Figure 6:**
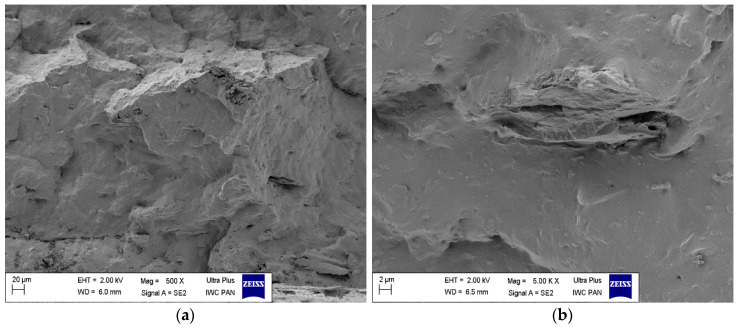

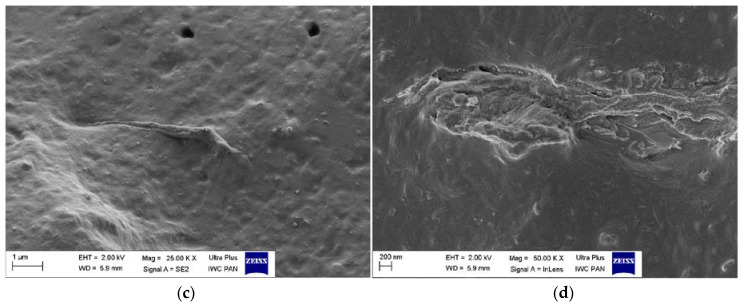
SEM images of biocomposites filled with 30 phr of horsetail under magnification: (**a**) 500× (**b**) 5.00× (**c**) 25.00× (**d**) 50.00×.

The morphology of biocomposites containing horsetail particles is shown in SEM images (Figure 6). The vulcanizates containing the filler were characterized by relatively good dispersion of horsetail particles in the elastomer matrix. Nevertheless, larger size particles or their aggregates were also present. It is worth emphasizing that even these large horsetail clusters were characterized by good rubber adhesion.

### 3.6. Rheometric Properties

**Figure 7:**
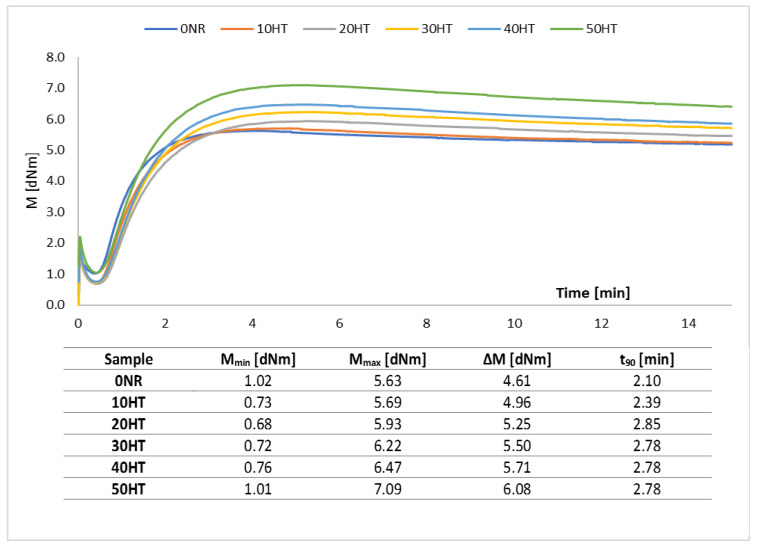
Rheometric curves and curing parameters of tested rubber mixtures.

The rheometric curves of the reference sample and horsetail-filled composites are shown in Figure 7. The minimum (M_min_) and maximum (M_max_) torque, as well as the increase in torque (ΔM) during rheometric measurements, were determined based on the obtained curves. The vulcanization time (t_90_) was also estimated, which is defined as the time after which a 90% increase in torque compared to the minimum value was observed.

The minimum torque is an indirect measure of the viscosity of mixtures. For the reference sample, the M_min_ value was 1.02 dNm. The viscosity of mixtures containing horsetail was lower, as evidenced by the reduction of the minimum torque compared to the 0NR sample. However, the M_min_ values depended on the amount of filler used. Generally, it could be stated that as the filler content increases, the material’s viscosity increases. The maximum torque recorded during rheometric measurements was higher for composites containing lignocellulosic material than for the unfilled mixture. The increase in M_max_ observed for horsetail-based composites was the greater the higher the filler content in the mixtures. The addition of rigid filler particles that are not deformed caused an increase in material stiffness manifested in an increase in the maximum torque value. The difference between the maximum and minimum torque (ΔM) was greater for horsetail filled systems. In elastomer technology, the increase in torque during rheometric measurements can be considered as an indirect indicator of the crosslinking density [48]. According to the data from Figure 7, the difference in torque, and thus the crosslinking density, was constantly increasing with the increase in the mass share of horsetail. These results might indicate that the filler also participates in crosslinking reactions, contributing to the development of the physical spatial network of composites. Finally, when analyzing the vulcanization time of the compositions, it should be noted that the addition of filler in the form of horsetail did not significantly affect its value. Curing time was in the range of 2.10–2.85 min.

### 3.7. Thermogravimetric Analysis (TGA) of the Composites

**Figure 8:**
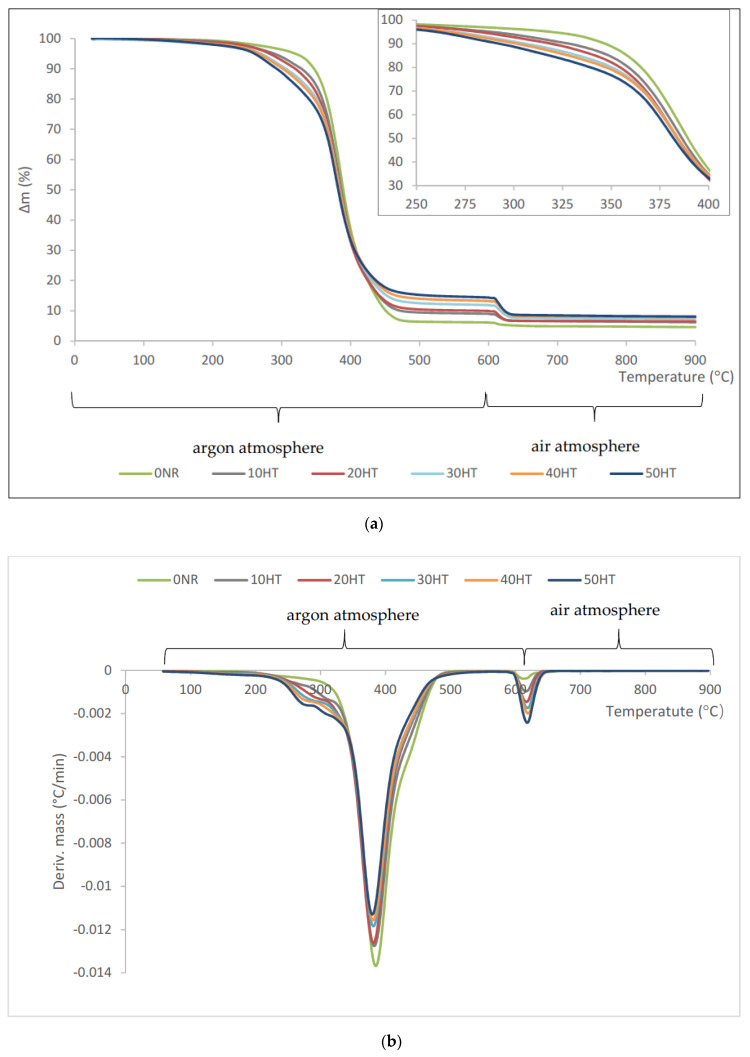
(**a**) TG and (**b**) DTG curves of natural rubber (NR) vulcanizates containing horsetail.

Thermal decomposition of composites containing horsetail proceeded in several stages (Figure 8). In the first stage of the process, the pyrolysis of natural rubber occurred in an argon atmosphere, which was accompanied by thermal decomposition of organic components (crosslinking agent and filler). The initial decomposition temperature (T_5%_) was lower for vulcanizates containing horsetail and corresponded to its content (Table 3). The more lignocellulosic material the lower the T_5%_ value. For the reference vulcanizate, a 5% weight loss occurred at 320 °C, while the addition of 10 phr horsetail to natural rubber caused a decrease in the T_5%_ value to 290 °C. The composite containing the largest amount of horsetail was characterized by the lowest thermal stability. The T_5%_ temperature for this composite was 260 °C. The addition of a filler to natural rubber undoubtedly influenced the intensity of the thermal decomposition process. On the DTG curves (Figure 8b) it is clearly seen that the composites containing the lignocellulosic material began to degrade at lower temperatures. In turn, the degradation process was faster in the temperature range of 230–340 °C for the reference composite. At T_50%_, no such significant changes in the mass loss were observed. The solid residue after the pyrolysis process at 600 °C was higher for filled composites compared to the reference sample. The greater the degree of filling of the rubber, the more solid residue was observed. This was due to a significant amount of inorganic substances found in the field horsetail, which have not decomposed in this temperature range. After changing the gaseous medium to air, at a temperature above 600 °C, the pyrolysis residue of rubber and some horsetail compounds were burnt. The mass losses Δm (600–900 °C) were greater, the more filler was in the composite. The combustion residue at 900 °C (R_900_) for the reference sample was 4.2, while for composites the R_900_ value increased in proportion to the horsetail content in the vulcanizate. The increased amount of residue after combustion of the filled composites might be due to the presence of silica in the horsetail, which remained in the ash.

### 3.8. Mechanical Properties

The mechanical properties of biocomposites and the reference system were determined in static tensile tests, the results of which are shown in Table 4. The lignocellulosic filler added to natural rubber contributed to significant changes in the modules at 100%, 200%, and 300% of elongation. An increase in SE_100_, SE_200_, SE_300_ values was observed for all filled composites compared to the free-filler vulcanizate. The values of all modules (SE_100_, SE_200_, SE_300_) increased with increasing content of filler in natural rubber. This demonstrates the large strengthening effect in the low-range deformations obtained for horsetail composites. For vulcanizates containing 40 phr of horsetail, stress values at 100%, 200%, and 300% have doubled. For larger strains that cause breakage, the largest reinforcement effect was observed for composite filled with the 10 phr of HT. The addition of larger amounts of horsetail caused a gradual decrease in the value of TS. Nevertheless, even for the highest filler content, the tensile strength did not deteriorate compared to the reference sample. The value of elongation at break of the composites corresponded to the amount of horsetail added and decreased with the increasing content of the filler.

### 3.9. Aging Processes

The presence of unsaturated structures (double bond >C=C<) in the hydrocarbon chain of natural rubber increases its susceptibility to degradation under the influence of UV radiation, heat, water, ozone, and oxygen [49]. In order to determine the resistance of produced composites to external factors, vulcanizates were subjected to the simulation of aging processes. Ultraviolet (UV), weather (W) and thermo-oxidative (TO) aging were carried out. The assessment was based on changes in crosslinking density, color and mechanical properties.

#### 3.9.1. Effect of Aging on the Crosslinking Density of NR Vulcanizates

**Figure 9:**
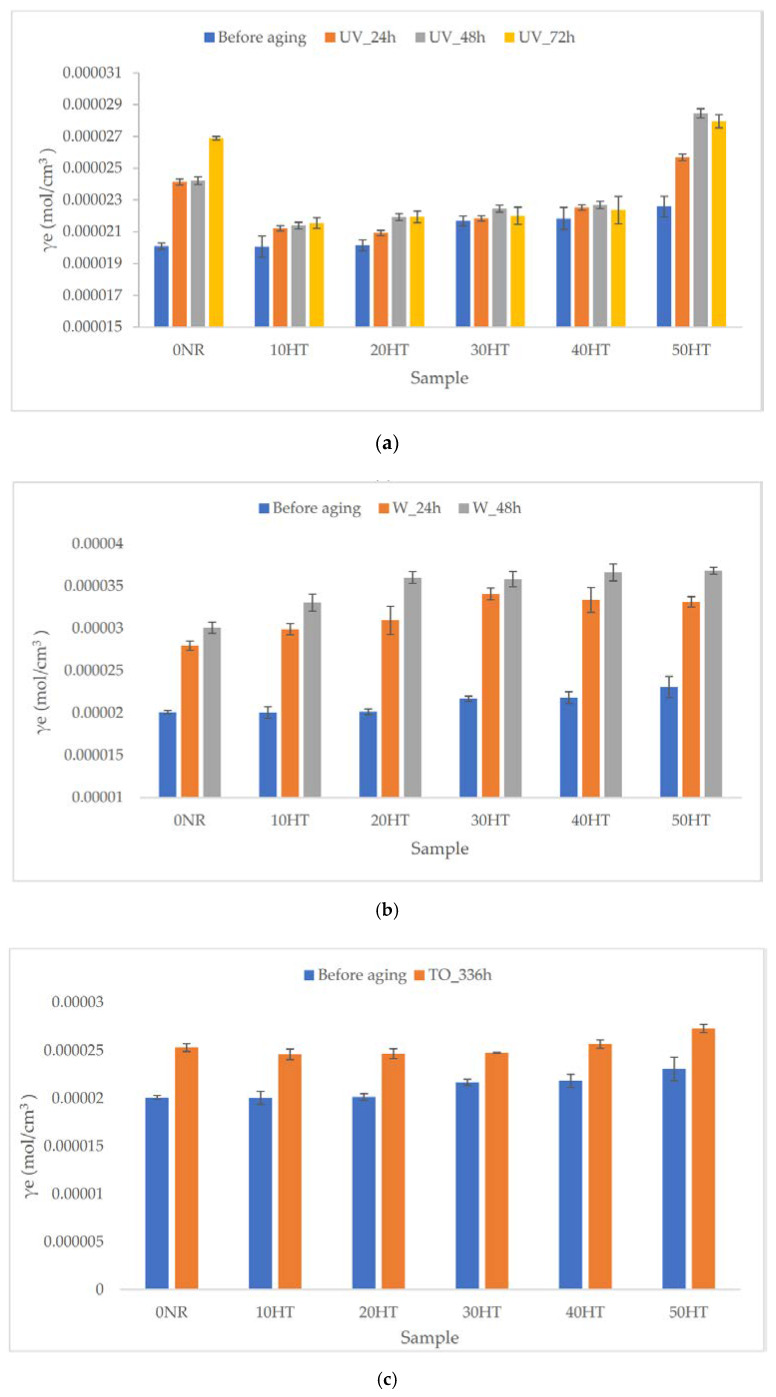
The crosslinking density of vulcanizates before and after simulated aging processes (**a**) UV, (**b**) weather, (**c**) thermo-oxidative.

Figure 9 shows the changes in the crosslinking density of vulcanizates before and after simulation of aging processes (a) UV, (b) weather, and (c) thermo-oxidative.

Data presented in Figure 9a indicated an increase in the crosslinking density of horsetail-based composites compared to the unfilled system. The increase in the number of nodes in the biocomposite spatial network correlated with the increase in the share of lignocellulosic material in the vulcanizate. The results obtained are consistent with the rheometric properties of the rubber mixtures discussed earlier.

The simulated UV aging process of vulcanizates contributed to a further increase in the γ_e_ values. The highest increase in crosslinking density was noted for the reference system and the composite with the highest horsetail content. A significant increase in crosslinking density of composites containing 50 phr of horsetail after UV aging was noted. Perhaps the percolation threshold was exceeded and such horsetail content did not inhibit the aging of natural rubber so strongly. In the case of other composites, the internal spatial network did not change significantly. The impact of exposure time on the γ_e_ parameter value varied, however, in most cases an increase in crosslinking density was observed with the increase in exposure to UV radiation.

In the case of weather aging (Figure 9b), an increase in the γ_e_ value for vulcanizates after the crosslinking process was also visible. In contrast, the increase in new nodes of the "network structure" was much higher for vulcanizates containing lignocellulosic material compared to the reference system. The impact of aging on the expansion of the spatial network was observed. Exposure of vulcanizates resulted in an increase in crosslinking density. In addition, among the types of aging studied, weather aging caused the largest changes in γ_e_ values. 

The increase in crosslinking density for composites aged in thermo-oxidative conditions was at a similar level for all vulcanizates. Samples were characterized by an increase in γ_e_ value in the range from 0.52 × 10^−7^ to 0.30 × 10^−7^ mol/cm^3^ (Figure 9c).

In each case, regardless of the aging type, vulcanizates after simulation of aging processes were characterized by higher crosslinking density. During aging, there are also intermolecular reactions that cause an increase in the molecular weight of the polymer and the formation of chain molecules, which leads to an extension of the molecular weight distribution. Consequently, the flexibility of the molecules, segmental mobility, and swelling are reduced [50]. In addition, the elevated temperature could lead to further crosslinking reactions resulting in the expansion of the spatial network of vulcanizates and affecting the total number of connections (nodes) in the composite [51].

In the case of oxidative and thermo-oxidative degradation, the mechanism consists of the formation of free radicals on the natural rubber (NR) chain through the uptake of hydrogen [52]. The reactions take place in several stages:

- the reaction of a free radical with an oxygen molecule and formation of a peroxide radical (NR−O−O)
- isolation of a hydrogen atom from another polymer chain with hydroperoxide generation (NR−O−OH)
- decomposition of hydroxide into two new free radicals (NR−O and OH)
- radical recombination or disproportionation.

The results of these reactions are polymer embrittlement and cracking. Aging processes also lead to the activation of intermolecular reactions. As a result of their action, the molecular weight distribution is expanded, caused by an increase in the molecular weight of the polymer and the formation of other chain molecules. As a result, particle flexibility, segmental mobility, and swelling are reduced. This leads to an increase in crosslinked density, reducing the elasticity of composites and mechanical strength.

In turn, in the case of the photo-degradation mechanism [52], free radicals are produced directly by UV radiation. Then all subsequent reactions are similar to those from thermal-oxidative degradation. Photo-degradation also occurs as a result of breaking bonds between the rubber and the filler, contributing to a decrease in crosslinking density.

#### 3.9.2. Effect of Aging on the Mechanical Properties of NR Vulcanizates

Mechanical properties after simulation of aging processes were also examined. Data regarding tensile strength and elongation at break are given in Table 5, Table 6 and Table 7. Moreover, the aging factor (K) was calculated based on the strength values obtained. This factor is a measure of the change in strain energy of the sample due to the aging process. It allows quantitative estimation of changes in mechanical properties caused by degradation. The smaller the changes in the mechanical properties of vulcanizates as a result of the aging process, the higher the K values and, as a result, the better elastomer resistance to aging.

According to the data in Table 5, the effects of ultraviolet radiation caused the largest changes in mechanical properties for the reference sample. There was a reduction in tensile strength by about 2.7–3.0 MPa and elongation by up to 60%. These changes resulted in the lowest aging rate being obtained. In contrast, the addition of horsetail improved the resistance to the aging of natural rubber under the influence of UV radiation. The increasing amount of filler caused smaller and smaller changes in the values of mechanical strength, and hence a higher K value. For example, for a composite containing 50 phr horsetail, TS changes of 0.7–1.5 MPa were noted compared to the unaged vulcanizate, while the changes in Eb values differed not more than 40%. The time of exposure to ultraviolet radiation slightly affected the deterioration of mechanical properties.

The weather aging process resulted in a significant decrease in tensile strength and elongation at break of composites with the addition of horsetail (Table 6). This was probably the result of a significant increase in crosslinking density. In the case of this type of aging, the results obtained indicate the reverse situation that occurred for UV aging. After the weathering process, the reference sample showed the highest resistance to aging. The application of lignocellulosic material contributed to the decrease in the value of the K coefficient, which decreased with increasing filling. In addition, a negative effect of longer aging times on strength parameters was observed.

The tested vulcanizates showed different resistance to thermo-oxidative aging (Table 7). Similar to the case of crosslinking density, the changes in mechanical properties were small. The highest value of the K coefficient was characterized by a composite containing 10 phr of horsetail. A further increase in the amount of horsetail resulted in a reduction of both TS and Eb values, which in consequence decreased the vulcanizates’ resistance to thermo-oxidative aging. The lowest K value was noted for the material filled with 50 phr horsetail (0.75). A slightly higher value of the aging factor was obtained for unfilled natural rubber—0.78.

Summing up the collected data of mechanical properties and crosslinking density before and after aging, a positive effect of horsetail addition on the composite’s resistance to UV radiation was proven. This may be the effect of active ingredients (polyphenolic acids or flavonoids) present in field horsetail. The absorption of UV radiation by the filler was confirmed by UV-VIS spectroscopy, where the absorption peak in the wavenumber range corresponding to ultraviolet radiation was observed.

In terms of mechanical properties, changes due to weather aging tend to be unfavorable. In this case, significant decreases in strength parameters were observed after stimulation of weathering processes. In this aging, except for radiation and high temperature, high humidity conditions were used. Field horsetail as a lignocellulose material is characterized by high water absorption, this phenomenon adversely affects the interfacial interactions (filler-polymer) thereby influencing the properties of the material. In addition, a similar situation occurred for high-filled composites subjected to thermo-oxidative aging. Prolonged exposure to elevated temperature might have led to thermal degradation of the lignocellulosic material, contributing to significant changes in properties.

#### 3.9.3. Color Change of NR Vulcanizates after Aging Processes

**Figure 10:**
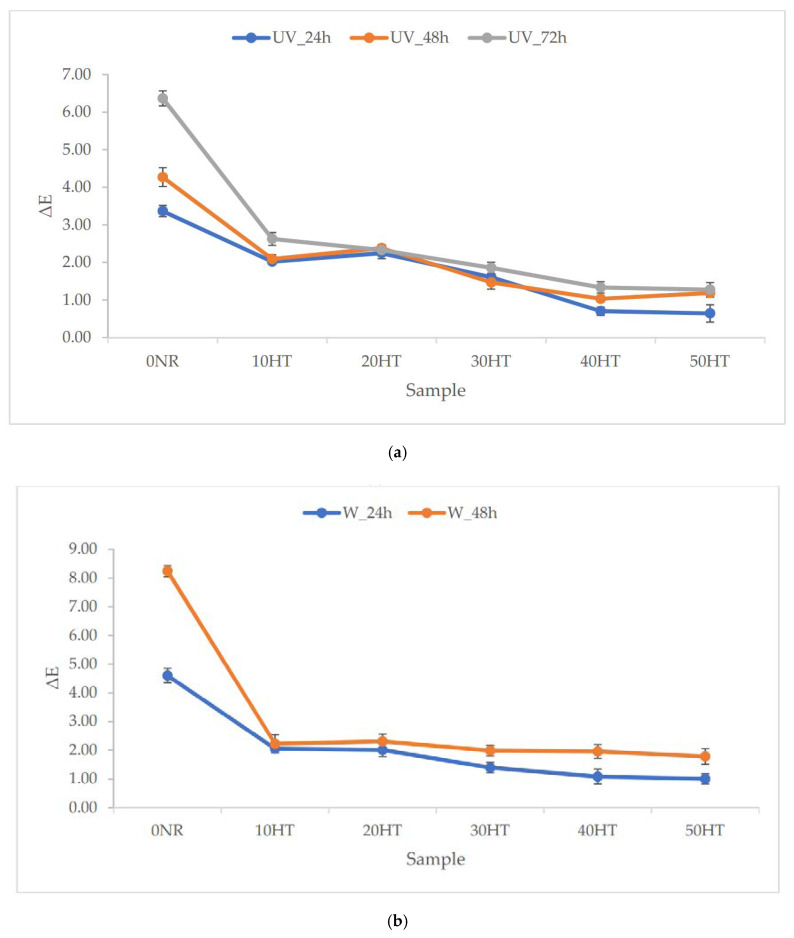

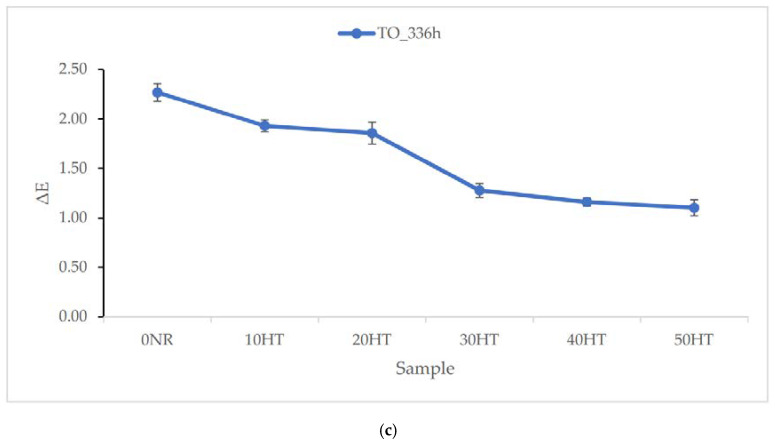
The total color change (ΔE) of the samples in the CIE-Lab color system as a result of aging processes (**a**) UV, (**b**) weathering, (**c**) thermo-oxidative.

Another property of vulcanizates that was tested before and after aging is the total color change (ΔE) of the samples in the CIE-Lab color system. Aging often leads to color changes in samples. From the data presented in Figure 10, it appeared that the biggest changes were caused by UV radiation and weather aging factors. In both cases, the exposure time to the aging stimulus was also important. The longer the sample was subjected to aging, the more visible the change in color. The least resistant to color change was the unfilled vulcanizate, for which the ΔE value even reached 8.24 and 6.37 for aging W_48h and UV_72h. The addition of filler allowed in each case to limit color changes. The best effect of color stabilization by adding a filler was observed for samples aged with UV radiation. Aged vulcanizates containing the largest amounts of horsetail were characterized by ΔE values even below 1. This fact can also be explained by the absorption of UV radiation by the filler components. The smallest color changes were noted for samples aged under thermo-oxidative conditions.

In the case of UV aging, a clear improvement in resistance to UV radiation was obtained for vulcanizates containing horsetail. Field horsetail consists of, among others, phenolic acids and flavonoids. These compounds have antioxidant activity. Flavonoids can absorb the most energetic solar wavelengths (UV radiation), they inhibit and then suppress reactive oxygen species (ROS). Due to the specific effects of horsetail components, this material has anti-aging effects (particularly on UV radiation) in elastomeric composites. This was confirmed based on results obtained in the tests of cross-linking density, tensile strength and color stability of vulcanizates.

## 4. Conclusions

The study aimed to characterize field horsetail as a new filler for elastomer composites. Until now, this plant has been used mainly as a medicinal herb and source of biogenic silica. Thermal horsetail analysis showed that biomass degradation begun at 170 °C, which provides sufficient conditions for the vulcanization process. On the horsetail UV-VIS spectrum, a clear peak of absorption in the UV range was recorded. This could indicate the presence of active antioxidant compounds such as flavonoids and phenolic acids in the tested material. The analysis of selected heavy metals in the tested materials showed satisfactory results, especially in the context of further research. Potentially, they can be used for example as ecological sound-absorbing barriers [53]. Ground horsetail was used as a filler for natural rubber. The application of this lignocellulosic material has contributed to an increase in the crosslinking density of NR vulcanizates, as well as to the improvement of their mechanical properties. The effect of the addition of horsetail on the aging processes of vulcanizates in various conditions (thermo-oxidative, UV, and weather aging) was also investigated. The assessment was based on differences in color, crosslinking density and mechanical properties of materials. The use of weather aging simulations resulted in the largest changes in the biocomposite spatial network and a significant reduction in the strength parameters of filled vulcanizates. However, in the case of UV aging, a clear improvement in resistance (in terms of mechanical strength) to UV radiation was obtained for vulcanizates containing horsetail. This is probably the effect of the active substances contained in horsetail limiting photooxidative reactions. Moreover, the analysis of materials subjected to thermo-oxidative aging also proves the positive effect of the addition of horsetail (up to 40 phr) in the context of maintaining mechanical properties at a sufficiently high level. Finally, composites containing *Equisteum arvense* as a natural filler were characterized by increased color stability regardless of the type of aging process simulated

## Figures and Tables

**Table 1:** Formulations of rubber composites.

Sample	0NR	10HT	20HT	30HT	40HT	50HT
HT (phr)	-	10	20	30	40	50
NR (phr)	100					
ZnO (phr)	5					
MBT (phr)	2					
SA (phr)	2					
S (phr)	1					

phr means parts per hundred parts of rubber

**Table 2:** Metal(II) concentrations in horsetail and natural rubber vulcanizates.

Samples	Co(II) (mg/kg)	Ni(II) (mg/kg)	Cd(II) (mg/kg)	Pb(II) (mg/kg)
0NR	0.16	0.88	0.41	27.07
10HT	0.22	0.96	0.42	24.17
20HT	0.25	1.04	0.40	21.91
30HT	0.32	1.22	0.39	21.43
40HT	0.32	1.23	0.36	17.96
50HT	0.35	1.18	0.35	16.71
Horsetail pure	0.80	2.04	0.24	0.86

**Table 3:** Decomposition temperatures at 5% (T_5%_), 50% (T_50%_), total mass loss during decomposition of vulcanizates and residue after combustion (R_900_).

Sample	T_5%_ (°C)	T_50%_ (°C)	Δm (25–600 °C) (%)	Δm (600–900 °C) (%)	R_900_ (%)
	Argon Atmosphere			Air Atmosphere	
0NR	320	389	6.2	1.5	4.6
10HT	290	386	9.0	2.8	6.2
20HT	282	384	10.0	3.4	6.5
30HT	270	384	11.9	4.4	7.5
40HT	267	384	13.3	5.3	7.9
50HT	260	382	14.4	6.3	8.1

**Table 4:** Mechanical properties of biocomposites: the modules at 100%, 200%, and 300% of elongation (SE_100_, SE_200_, SE_300_), tensile strength (TS) and elongation at break (Eb).

Sample	SE_100_ (MPa)	SE_200_ (MPa)	SE_300_ (MPa)	TS (MPa)	Eb (%)
0NR	0.75 ± 0.03	1.12 ± 0.05	1.53± 0.03	12.6 ± 0.2	712 ± 7
10HT	0.87 ± 0.03	1.37 ± 0.03	1.90 ± 0.04	16.1 ± 0.3	754 ± 8
20HT	1.06 ± 0.04	1.69 ± 0.03	2.32 ± 0.07	15.2 ±0.4	720 ± 6
30HT	1.32 ± 0.05	2.05 ± 0.04	2.78 ± 0.02	14.6 ± 0.3	709 ± 9
40HT	1.44 ± 0.02	2.20 ± 0.05	2.98 ± 0.06	13.2 ± 0.3	678 ± 8
50HT	1.83 ± 0.04	2.69 ± 0.05	3.69 ± 0.04	12.7 ± 0.5	611 ± 8

**Table 5:** The changes in mechanical properties before and after simulated UV aging processes and aging factor (K) for biocomposites. Standard deviations did not exceed: TS ± 0.5 MPa, Eb ± 10%, K ± 0.05.

No Aging			UV_24h			UV_48h			UV_72h		
Sample	Before Aging		After Aging		K	After Aging		K	After Aging		K
	TS (MPa)	Eb (%)	TS (MPa)	Eb (%)		TS (MPa)	Eb (%)		TS (MPa)	Eb (%)	
0NR	12.6	712	9.9	653	0.72	9.6	648	0.69	9.6	645	0.69
10HT	16.1	754	12.8	719	0.76	12.6	719	0.74	12.3	708	0.72
20HT	15.2	720	13.0	718	0.85	12.3	710	0.80	12.2	711	0.79
30HT	14.6	709	13.0	706	0.88	11.3	706	0.77	11.3	705	0.77
40HT	13.2	678	12.2	693	0.95	10.6	680	0.80	10.9	655	0.80
50HT	12.7	611	11.7	597	0.90	12.1	588	0.91	11.2	575	0.83

**Table 6:** The changes in mechanical properties before and after simulated weather aging processes and aging factor (K) for biocomposites. Standard deviations did not exceed: TS ± 0.4 MPa, Eb ± 8%, K ± 0.04.

No Aging			W_24h			W_48h		
Sample	Before Aging		After Aging		K	After Aging		K
	TS (MPa)	Eb (%)	TS (MPa)	Eb (%)		TS (MPa)	Eb (%)	
0NR	12.6	712	11.8	654	0.86	11.3	642	0.81
10HT	16.1	754	13.2	665	0.72	10.0	619	0.51
20HT	15.2	720	9.7	640	0.57	7.1	620	0.40
30HT	14.6	709	8.0	602	0.46	7.1	601	0.41
40HT	13.2	678	6.8	602	0.46	6.7	598	0.45
50HT	12.7	611	7.4	558	0.53	7.1	557	0.51

**Table 7:** The changes in mechanical properties before and after simulated thermo-oxidative aging processes and aging factor (K) for biocomposites. Standard deviations did not exceed: TS ± 0.6 MPa, Eb ± 11%, K ± 0.05.

Sample	Before Aging		After Aging TO_336h		K
	TS (MPa)	Eb (%)	TS (MPa)	Eb (%)	
0NR	12.6	712	10.7	652	0.78
10HT	16.1	754	15.5	691	0.88
20HT	15.2	720	14.4	665	0.87
30HT	14.6	709	13.0	654	0.82
40HT	13.2	678	11.7	626	0.82
50HT	12.7	611	10.0	583	0.75
